# Machine learning and single cell RNA sequencing analysis identifies regeneration-related hepatocytes and highlights a Birc5-related model for identifying cell proliferative ability

**DOI:** 10.18632/aging.204775

**Published:** 2023-06-14

**Authors:** Yuan Du, Shuqin Jian, Xicheng Wang, Chao Yang, Hua Qiu, Kang Fang, Yehong Yan, Jun Shi, Jianfeng Li

**Affiliations:** 1 Department of General Surgery, The First Affiliated Hospital of Nanchang University, Nanchang, Jiangxi, China; 2 Department of Rehabilitation Medicine, The First Affiliated Hospital of Nanchang University, Nanchang, Jiangxi, China; 3 Institute for Regenerative Medicine, Shanghai East Hospital, School of Life Sciences and Technology, Tongji University School of Medicine, Shanghai, China; 4 Shanghai Engineering Research Center of Stem Cells Translational Medicine, Shanghai, China; 5 Shanghai Institute of Stem Cell Research and Clinical Translation, Shanghai, China; 6 Department of General Surgery, Ji’an Hospital of Shanghai East Hospital, School of Medicine, Tongji University, Ji’an, Jiangxi, China

**Keywords:** liver regeneration, Hmgb1, Birc5, hdWGCNA, machine learning

## Abstract

Background: Partial hepatectomy (PHx) has been shown to induce rapid regeneration of adult liver under emergency conditions. Therefore, an in-depth investigation of the underlying mechanisms that govern liver regeneration following PHx is crucial for a comprehensive understanding of this process.

Method: We analyzed scRNA-seq data from liver samples of normal and PHx-48-hour mice. Seven machine learning algorithms were utilized to screen and validate a gene signature that accurately identifies and predicts this population. Co-immunostaining of zonal markers with BIRC5 to investigate regional characteristics of hepatocytes post-PHx.

Results: Single cell sequencing results revealed a population of regeneration-related hepatocytes. Transcription factor analysis emphasized the importance of Hmgb1 transcription factor in liver regeneration. HdWGCNA and machine learning algorithm screened and obtained the key signature characterizing this population, including a total of 17 genes and the function enrichment analysis indicated their high correlation with cell cycle pathway. It is note-worthy that we inferred that Hmgb1 might be vital in the regeneration-related hepatocytes of PHx_48h group. Parallelly, Birc5 might be closely related to the regulation of liver regeneration, and positively correlated with Hmgb1.

Conclusions: Our study has identified a distinct population of hepatocytes that are closely associated with liver regeneration. Through machine learning algorithms, we have identified a set of 17 genes that are highly indicative of the regenerative capacity of hepatocytes. This gene signature has enabled us to assess the proliferation ability of *in vitro* cultured hepatocytes using sequencing data alone.

## INTRODUCTION

The liver is a multifunctional internal organ closely related to human health and performs several vital physiological functions, such as decomposition of blood toxins, metabolic storage, lipid/glucose metabolism, secretion of serum proteins [1]. Hepatocytes, as the primary cell type within the liver, are responsible for these crucial functions. Previous single-cell transcriptome studies have carefully drawn the molecular atlas of adult liver cells [2], however, effective systematic integration of single-cell transcriptome studies on hepatocytes are urgently needed during liver regeneration [3]. Taken together, In-depth understanding of the specific mechanisms and state changes of hepatocytes during regeneration deepen our understanding of liver disease, opening up novel strategies for patients across the spectrum of liver diseases.

The liver is an exceptional organ with an innate ability to regenerate following injury. Even after the removal of up to 70% of its mass, the remaining tissue can regenerate an original-sized liver within a few months [4]. Hepatocytes represent the major cell type in the liver, and over 98% of them contribute to damage repair. During post-hepatectomy acute liver injury, newborn hepatocytes arise from the self-replication of residual hepatocytes, highlighting the proliferation and division of mature hepatocytes as the fastest and most effective method of liver regeneration [1]. Notably, Single-cell RNA sequencing (scRNA-seq) is a powerful approach to explore cell structure and characterize cell identity [5], especially identifying different cell populations and explaining the heterogeneity or correlation between cells. In conclusion, liver regeneration is a critical topic of interest in clinical scenarios and cutting-edge cellular therapies for liver disease. Advancing our understanding of the molecular basis and exploring the source of the proliferation process at the single-cell level will provide valuable insights into therapeutic targets for patients of liver disease.

The present study aims to investigate the key signatures and cell signaling pathways involved in liver regeneration. Our research team aims to identify molecules that could potentially be used to treat patients experiencing liver failure. Furthermore, the identified genes may serve as potential biomarkers to predict the likelihood of spontaneous liver regeneration in patients with damaged liver tissue, bridging the gap between bench-side research and bedside clinical application.

## MATERIALS AND METHODS

### Data collection

The data collected in this study were mainly from the GEO database (https://www.ncbi.nlm.nih.gov/geo/), and all the sequencing data were related to PHx. Details of these data are provided in Table 1 [6–11]. The collection of partitioned genes was mainly from the references.

**Table 1:** Overview of the information of analyzed datasets.

**Dataset**	**Year**	**Species**	**Platform**		**Data type**	**Team**
GSE151309	2021	Mus musculus	GPL24247	Illumina NovaSeq 6000	scRNA-seq	Chembazhi UV, et al.
GSE158866	2020	Mus musculus	GPL24247	Illumina NovaSeq 6000	scRNA-seq	Chen T, et al.
GSE133213	2022	Mus musculus	GPL18480 GPL21103	Illumina HiSeq 1500 Illumina HiSeq 4000	Bulk RNA-seq	Belenguer, et al.
GSE215423	2022	Mus musculus	GPL24247	Illumina NovaSeq 6000	Bulk RNA-seq	Guo, et al.
GSE83598	2017	Mus musculus	GPL7202	Agilent-014868 Whole Mouse Genome Microarray 4x44K G4122F	Microarray	https://www.ncbi.nlm.nih.gov/geo
GSE4528	2007	Mus musculus	GPL339	[MOE430A] Affymetrix Mouse Expression 430A Array	Microarray	https://www.ncbi.nlm.nih.gov/geo
GSE167034	2022	Mus musculus	GPL1261	[Mouse430_2] Affymetrix Mouse Genome 430 2.0 Array	Microarray	Holland C, et al.
GSE158864	2020	Mus musculus	GPL17021	Illumina HiSeq 2500	Bulk RNA-seq	Chen T, et al.
GSE212692	2023	Mus musculus	GPL21103 GPL24247	Illumina HiSeq 4000 (Mus musculus) Illumina NovaSeq 6000 (Mus musculus)	scRNA-seq	Li L, et al.

### High dimensional WGCNA (hdWGCNA)

HdWGCNA can be used to perform weighted gene co-expression network analysis (WGCNA) in high dimensional data such as single cell RNA-seq [12]. HdWGCNA could be utilized to construct co-expression networks in a cell type-specific manner, identifies robust modules of related genes, and provides the biological context for these modules. In this article, we used hdWGCNA to explore the function of hepatocytes after hepatectomy. And finally screening characteristic genes of a hepatocyte regeneration subgroup. The R package version of hdWGCNA used in this study is 0.1.1.9010.

### Metacell algorithm

Single-cell transcriptome data have the characteristics of high background noise, low gene detection rate and sparse expression matrix. At present, genes with known functions and acquaintances can often be gathered together. Herein, obtaining metacell using the metacell algorithm [13, 14] can often better reflect the real expression of the cell. We performed the metacell analysis on the single cell sequencing data of hepatocytes to provide reliable cell data for further training and testing model for regenerative subpopulations.

### Construction of machine learning model

The use of single cell sequencing and key regulatory genes identified by hdWGCNA through machine learning may also help to improve understanding of liver regeneration. We use the Least Absolute Shrinkage and Selection Operator (Lasso) analysis and learn from seven machine learning algorithms to screen, get the appropriate algorithm and gene, so as to better characterize the state of hepatocytes. The seven machine learning algorithms were performed using the mlr3verse (version 0.2.7) package in R (https://CRAN.R-project.org/package=mlr3verse).

### Single sample gene set enrichment analysis (ssGSEA) and gene set enrichment analysis (GSEA)

To better understand the activation of the interested gene sets, ssGSEA algorithm in gene set variation analysis (GSVA, version 1.42.0) package [15] and the GSEA pipeline, which could uncover the detailed pathways of interest [16], were utilized. And clusterProfiler package (version 1.42.0) [17] and fgsea package (version 1.42.0) [18] were adopted to better visualize the interested and enriched pathways, including Gene Ontology (GO) and KEGG signalings. We used GSEA and fgsea analyses to understand the potential role of Birc5 in liver regeneration.

### Pseudo-time analysis

Monocle2 algorithm (version 2.22.0) [19] can clearly see the changing trend of up-regulation and down-regulation and even find hidden change patterns: We used trajectory analysis to in-depth explore the process of complete release of regeneration ability from mature hepatocytes to hepatectomy after 48h. And the changing expression of 17 key genes with time series was depicted.

### Cellular communication analysis

Cellular communication plays important roles in diseases. CellChat package (version 1.0.0) makes more detailed modifications to the signaling mechanism to recognize different levels of signal changes [20]. Using cell communication analysis, we explored the interaction of regeneration-related subsets with other cell types and compared them with other hepatocyte populations, as well as the differences and connections between hepatocytes and other non-essential cells during the current regeneration process. We stochastically selected 4000 hepatocytes for running the pipeline. Other parameters were set as default.

### Analysis of transcriptional factor regulatory network

Single-cell regulatory network interference and clustering (SCENIC, version 1.2.4) [21] is a gene regulatory networks (GRNs) algorithm specially developed for single cell data. SCENIC introduced the gene co-expression network deduced by transcription factor (TF) motifs, thereafter identifying high-reliability GRNs dominated by TFs. By using the SCENIC algorithm to explore the specific transcription factors of regeneration-related subpopulations compared with other subpopulations, we further revealed the possible relationship between Hmgb1 and liver regeneration.

### Partial hepatectomy liver model (PHx)

6~8weeks adult mice were housed in 12h light-dark cycle with standard diet. 2/3rd partial hepatectomy (PHx) procedure was implemented according to previously protocol [22]. Briefly, an 8~10mm incision was made on the skin below mediastinum with anesthetized (2% tribromoethanol, intraperitoneal injection), left lateral lobe and the median lobe were ligated by a 4-0 silk thread, and the lobes were resected just above the knots. Following this, peritoneum and skin were then closed with 6-0 sutures, and then mice were fed with normal saline solution and allowed to recover on a pre-warmed pad. Sham mice that underwent a similar surgical intervention except for the actual resection of the liver sections.

### Liver function test

Retro-orbital punctures were implemented to collect peripheral blood from mice into vacuum blood collection tubes. The upper layer of serum was isolated and centrifugation at 12000g for 8 min and stored at −80° C for further measurement. Serum levels of ALT and AST were performed at third-party inspection structure (Yuxiu Biotechnology, Shanghai, China).

### Tissue embedding and immunofluorescence staining

Tissue was collected in PBS and washed for 3 times, and then split into 10~15 mm size, fixed in 10% neutral buffered formalin for 48~72 h and embedded in paraffin. 3~4 μm thick sections were cut, deparaffinized in xylene, rehydrated and antigen retrieved in Tris-EDTA buffer (pH=9.0). The sections were washed with PBST (PBS+1%Tween20) and blocked for 1h at room temperature in blocking buffer (10% normal donkey serum+3% BSA in PBS), followed by primary antibody incubation overnight at 4° C. The next day, tissues were washed with PBST for 3times and incubated with fluorescence-conjugated secondary antibodies (Abcam) for 30~45 minutes at 37° C and washed with PBST again, mounted with mounting medium containing DAPI (Abcam). The used antibody included: rabbit anti-GS (Abcam, 49873, 1:1000 dilution), mouse anti-CDH1(Biosciences, 610181, 1:50 dilution), rabbit anti-Survivin (Abconal, A1551, 1:50 dilution), rabbit anti-Ki67(Abbomax, 500-1874, 1:200 dilution), mouse anti-β-catenin (Biosciences, 610153, 1:400 dilution). For continuous labelling of DNA synthesis post-PHx, EDU solution (Yeasen, 40284ES50, 0.5~1mg/ml dilution) was intraperitoneal injection every 12h before sacrifice within the first 3 days post-PHx. Images were acquired using Leica confocal microscope (Leica+STED+3X). Images were analyzed by the Image J software.

### Quantitative rt-PCR

After grinding liver tissue, Total RNA was extracted using Trizol reagent (Thermofisher, MA, USA) and reverse-transcribed into cDNA with 5×PrimeScriptTM RT Master Mix ((Takara Bio Inc., Shiga, Japan). 2×SYBR Green qRT-PCR Master Mix (Selleck, Selleck) was used to implement qRT-PCR. Target gene expression was uniformized according to the level of GAPDH expression in different samples and calculated using the 2^−ΔΔCt^. The primer sequences were shown in Table 2.

**Table 2:** Primers for all 17-genes identified by machine learning algorithms.

**Gene**	**Forward primer (5′-3′)**	**Reversed primer (5′-3′)**
Mus-Birc5	GAGGCTGGCTTCATCCACTG	CTTTTTGCTTGTTGTTGGTCTCC
Mus-Ssr2	TGTTAGCCGTCAGTCAAGCAG	GGAGCCGACGTTGTAGATGTT
Mus-Gstm3	CCCCAACTTTGACCGAAGC	GGTGTCCATAACTTGGTTCTCCA
Mus-Tmem53	TGGTCCTGGCGATAGCAAC	TACCATACGTTCCACATCCCT
Mus-Ube2c	CTCCGCCTTCCCTGAGTCA	GGTGCGTTGTAAGGGTAGCC
Mus-Cdk1	AGAAGGTACTTACGGTGTGGT	GAGAGATTTCCCGAATTGCAGT
Mus-Psenen	GTGTTTCGGATGTCCCTCTACC	CAGCTCCCTCTTACGCTGG
Mus-Spcs1	GCCCACCCAGATGGATTACAA	GCCACGTACCCGTAGATAAACC
Mus-Apom	TAACTCCATGAATCAGTGCCCT	CCCGCAATAAAGTACCACAGG
Mus-Reep5	GGTTCCTGCACGAGAAGAACT	GAGAGAGGCTCCATAACCGAA
Mus-Hmgb1	GGCGAGCATCCTGGCTTATC	GGCTGCTTGTCATCTGCTG
Mus-Cdc20	TTCGTGTTCGAGAGCGATTTG	ACCTTGGAACTAGATTTGCCAG
Mus-Tmem176a	GCCGGATGCTCATTGCTAAG	ATGGCCTATGTAGAGGGTTCC
Mud-GAPDH	AGGTCGGTGTGAACGGATTTG	TGTAGACCATGTAGTTGAGGTCA

### Statistical analysis

Statistically significant difference is estimated by using Student’s 2-tailed t test. *P* values of less than 0.05 were considered statistically significant. The correlation analysis was performed using the cor function in R (R version 4.1.0).

## RESULTS

### Single cell RNA-sequencing analysis of liver regeneration at 48h after partial hepatectomy (PHx)

**Figure 1:**
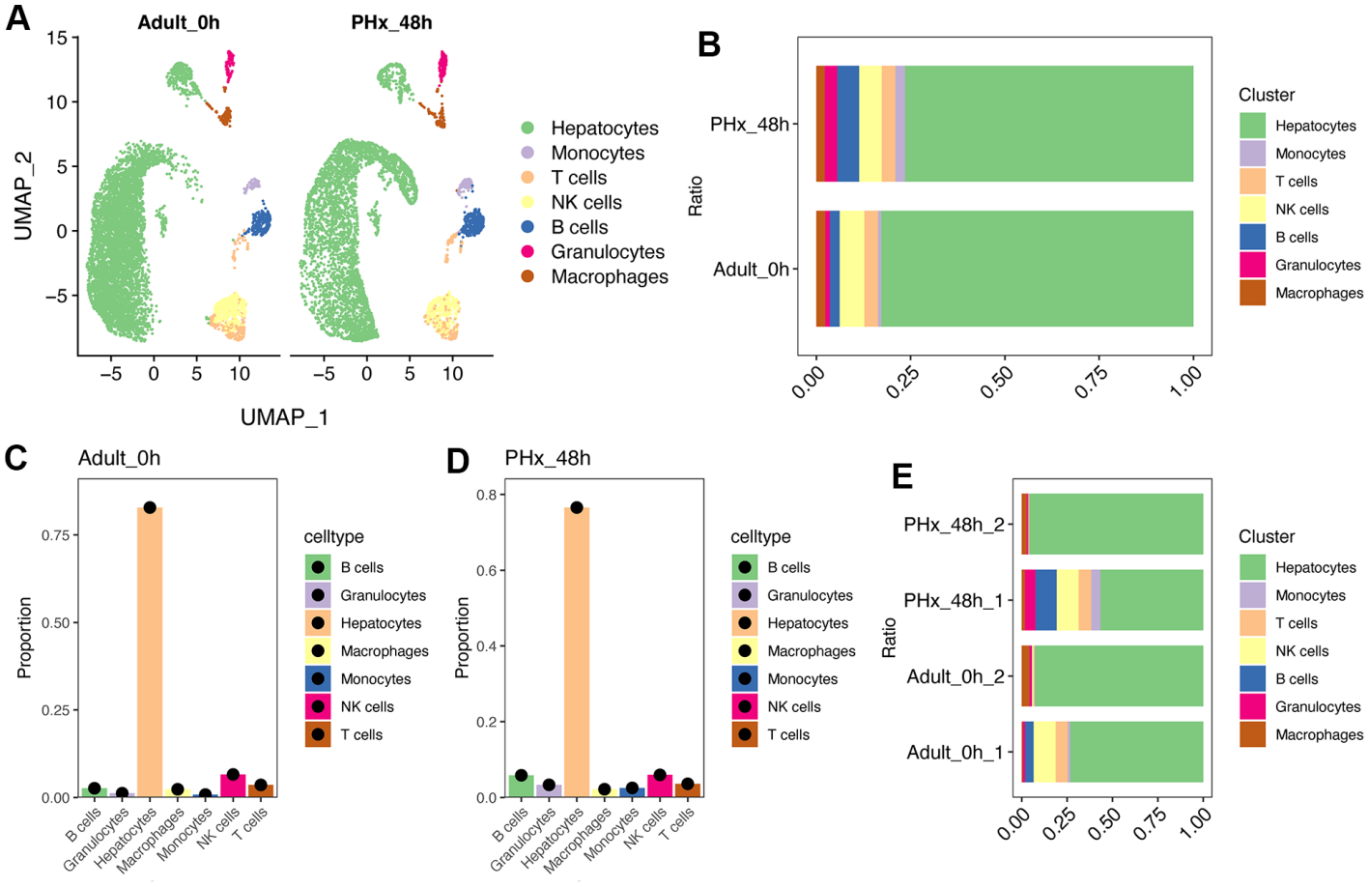
**Single cell analysis of cell proportion changes of liver regeneration after PHx.** (**A**) UMAP plot shows the distribution of different cell types of PHx livers. (**B**) Cell proportion of seven cell types in PHx livers. (**C**) Bar plot indicates the cell proportion of all seven cell types in liver at 0h after PHx. (**D**) Bar plot indicates the cell proportion of all seven cell types in liver at 48h after PHx. (**E**) Cell proportion of seven cell types in PHx livers. The cell proportion in four samples of liver was showed.

Liver has great power of regeneration function and the proliferative ability will reach peak at 48h after partial hepatectomy (PHx) [6, 7]. Therefore, we tended to explore changes between the proliferative state and quiescent state of liver organ. Here, we collected two scRNA-seq data containing mouse liver cells from adult_0H and PHx_48h groups (Figure 1A). After removing batch effect and with stringent criteria, we had narrowed and filtered 15065 liver cells, and finally 13032 liver parenchymal and nonparenchymal cells for subsequent analyses (Supplementary Figures 1, 2). Clearly, we could find that different cell types were distributed and clustered separately using reduction analysis and UMAP plot visualization (Figure 1A). Then, the proportion of different cell types were showed in Figure 1B–1E. Interestingly, the obtained non-parenchymal liver cells will increase at 48h after PHx both in the two 48h scRNA-seq samples (Figure 1E), indicating the involvement of cell-cell communication during regeneration.

### HdWGCNA analysis highlights the pink module for charactering the potential functions of regeneration-related clusters

**Figure 2:**
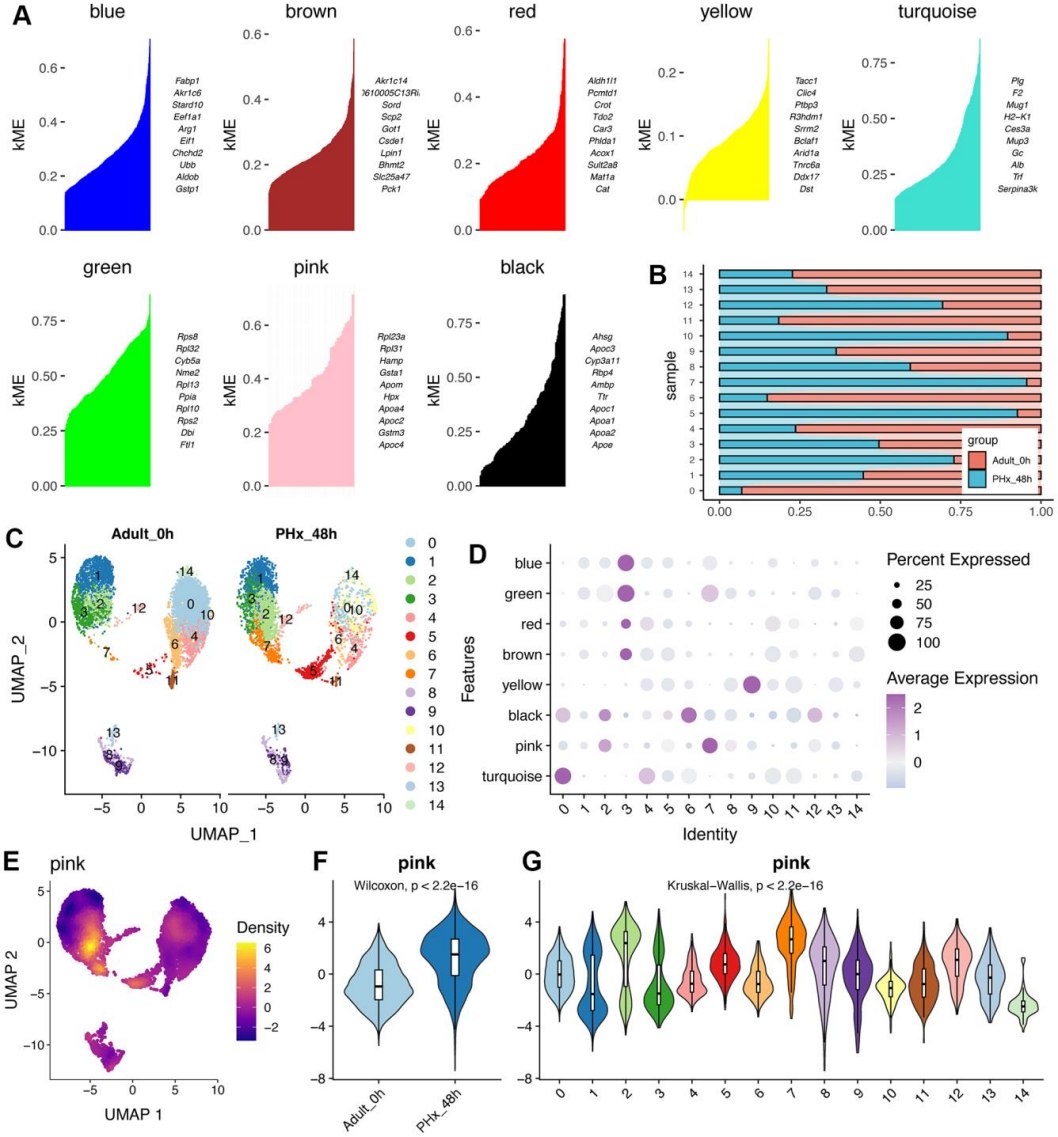
**HdWGCNA of hepatocytes reveals the crucial roles of pink module.** (**A**) Eight gene modules were obtained and the top huh gene were presented according to the hdWGCNA pipeline. (**B**) Cell proportion of hepatocyte clusters in different time points of PHx. (**C**) UMAP plot shows the distribution of 15 hepatocyte clusters in PHx 0h and PHx 48h. (**D**) Module activities in different hepatocyte clusters. The module score was estimated by hdWGCNA algorithm. (**E**) Expression density of the pink module in UMAP plot. The yellow indicates the highest activity score of the module in corresponding cells. (**F**) Violin plot displays the higher activity of pink module in PHx 48h mice. (**G**) Activity levels of pink module in 16 hepatocyte clusters.

High Dimension WGCNA pipeline was used to explore the potential functions of hepatocytes. The hdWGCNA was applied and the detailed information of gene modules was showed in Supplementary Figure 3. As showed in Figure 2A, eight gene modules were obtained and the top huh gene were presented following the hdWGCNA pipeline. GO and KEGG analyses showed that most of the eight gene modules had various functions (Supplementary Figure 4). Parallelly, dimension reduction and subcluster analysis of hepatocytes was also conducted and 15 subclusters of hepatocytes were gained (Figure 2B, 2C). Intriguingly, we could find that cluster2, 5, 7, 10 and 12 were tended to be up-regulated in PHx liver tissues, especially cluster7 (Figure 2B, 2C). Then, we have evaluated the module scores in these clusters (Figure 2D and Supplementary Figure 3D). Of interest, the pink module was highly activated mainly in cluster7 (Figure 2E). More, the activation score of the pink module was also higher in PHx group rather than adult normal liver group (Figure 2F). Additionally, the pink module was also activated in cluster2 (Figure 2G). Thus, we considered that the pink module was related to the PHX-related hepatocyte clusters.

### Machine learning algorithms reveals the regeneration-related model and hub features

Considering that the genes in pink module might be pivotal and capable of distinction regeneration-related hepatocytes from other hepatocytes, we tried to construct a reliable model for identifying regeneration-related hepatocyte clusters in single cell levels. Single cell data have great noise, which might affect the robustness of the model. The metacell algorithm make it possible for similar single cells to cluster together. And rather than biological difference, distinctions between single cell and single cell in a “metacell” are likely only due to the technical effect [13, 14]. Therefore, we changed single cell data into meta-cell data, which could avoid or reduce the data noise. As a result, we obtained 117 regeneration-related meta-hepatocytes and 377 other-hepatocytes. Then, all the meta-hepatocytes were divided into training cohort and testing cohort according to the sample source. Thus, in the training cohort, we had 61 regeneration-related meta-hepatocytes and 229 other-meta-hepatocytes. While in the testing cohort, we had 56 regeneration-related meta-hepatocytes and 148 other-meta-hepatocytes.

**Figure 3:**
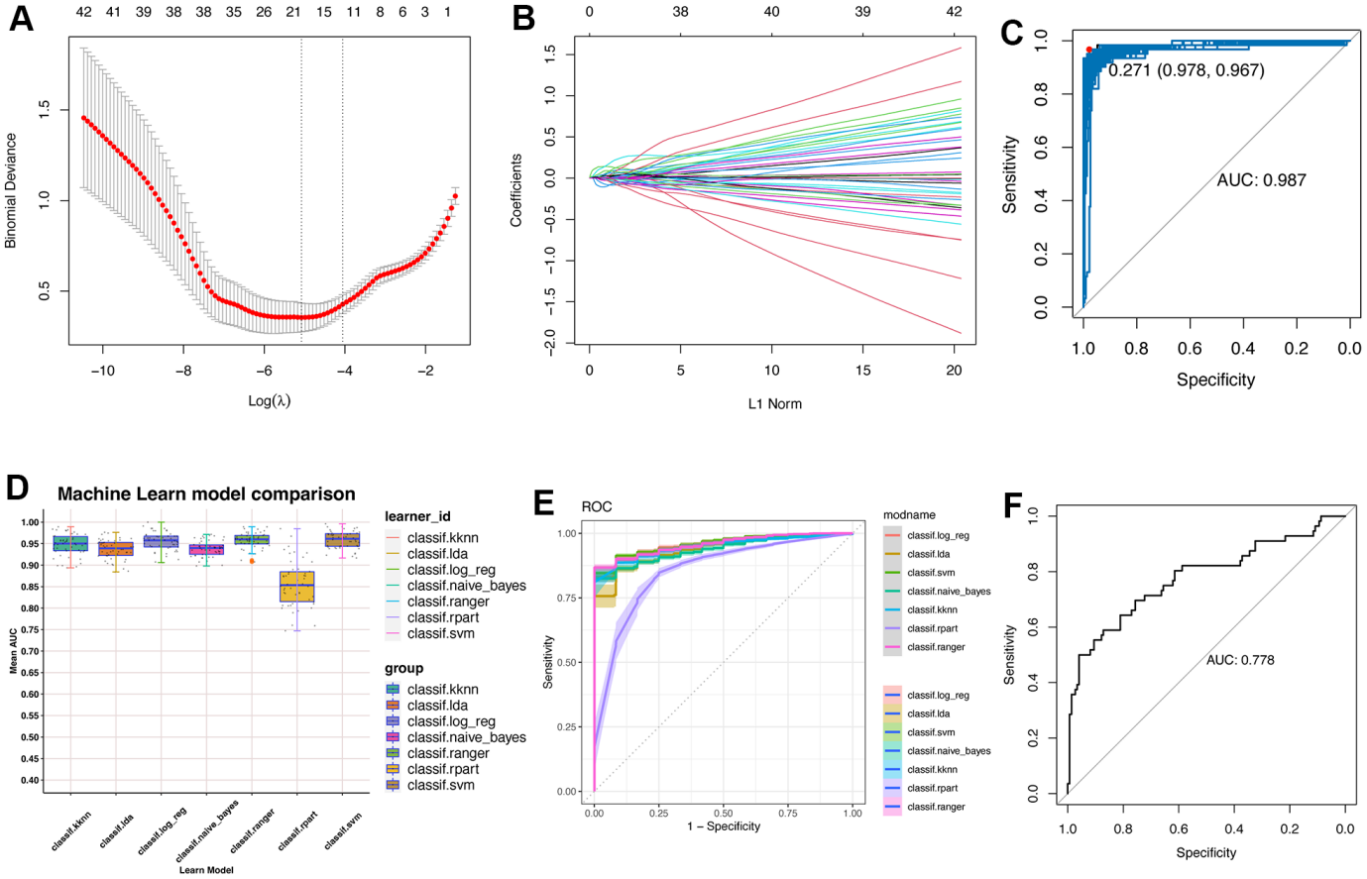
**Machine learning constructs model for predicting the regeneration-related hepatocytes.** (**A**) Lasso algorithm for selection features for regeneration-related hepatocytes. (**B**) Coefficient changes of the selected features using lasso algorithm. (**C**) Bootstrap algorithm for internal validation of the constructed model. (**D**) Machine learning algorithms for building the regeneration-related hepatocyte model. Seven machine learning algorithms were used in the mlr3verse (version 0.2.7) package in R (https://CRAN.R-project.org/package=mlr3verse). (**E**) ROC values of all seven algorithms were showed in training cohort. (**F**) ROC values of the SVM model in test cohort.

Then, we used LASSO algorithm for further filtered the genes in pink module (Figure 3A, 3B). And only 17 genes were left and considered powerful for constructing a classifier, including Ube2c, Ssr2, Gstm3, Tmem53, Cdc20, Tmem176a, Apoc4, Psenen, Pet100, Cdk1, Birc5, Rrm2, Hist1h2ap, Spcs1, Tuba1b, Apom and Reep5. Then, we used Boostrap algorithm for internal validation and result showed that the AUC was 0.987 (Figure 3C). With these 17 genes, we further tried to build a strong classifier for regeneration-related hepatocytes. Thus, seven machine learning algorithms were applied for estimating the performance (Figure 3D). Interestingly, data showed that the supporting vector machine (SVM) might have better performance in both internal and external cohorts (Figure 3E, 3F).

### Trajectory analysis of hepatocytes reveals the application of the regeneration-related 17-gene signature

**Figure 4:**
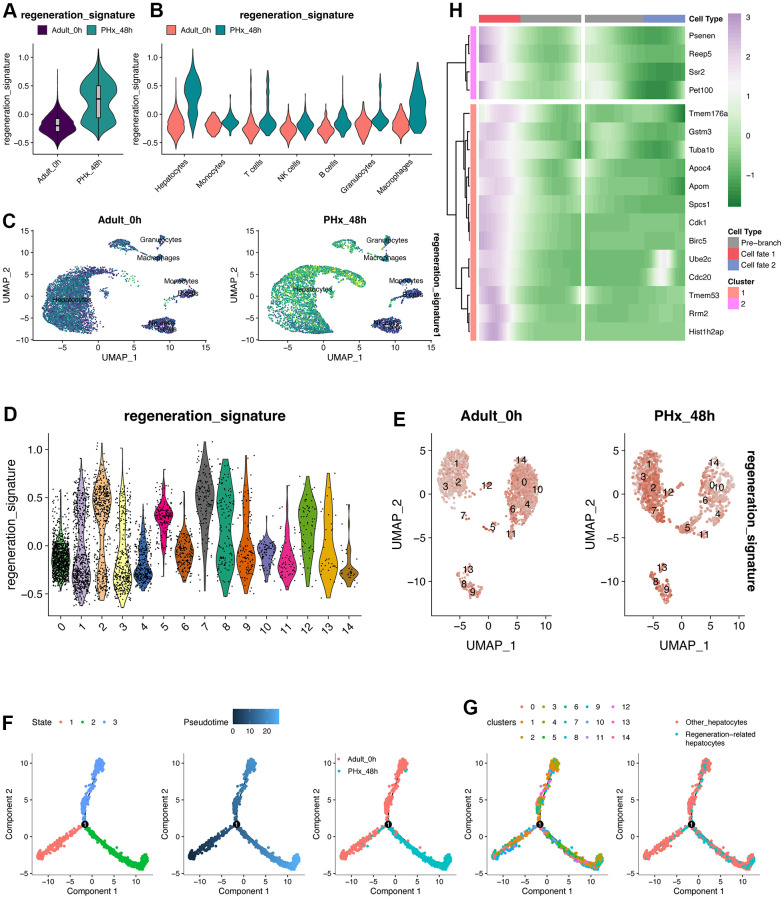
**Pseudotime analysis of hepatocytes.** (**A**) Regeneration signature score of pseudo-bulk expression of liver organ under PHx. (**B**) Regeneration signature score of pseudo-bulk expression of liver organ under PHx. (**C**) UMAP plot displays the distribution of the regeneration signature in all cell types of the PHx 0h and PHx 48h group. (**D**) Score of regeneration-related signature in all 15 hepatocyte clusters. The regeneration-related signature was composed of 17 machine-learning-identified genes. (**E**) UMAP plot depicts the expression changes of the regeneration-related signature. (**F**) Pseudotime analysis of the hepatocytes. Three states/branches were calculated by monocle2 package based on highly varied genes. (**G**) Pseudotime distribution of the 15 hepatocytes clusters. (**H**) Pseudotime heatmap plots the changes of 17 genes following the pseudotime changes.

After constructing the 17-gene classifier using metacell and machine learning algorithms, we tried to validate the identified 17 genes in single cell levels. As demonstrated in Figure 4A–4C, the regeneration 17-gene signature was higher in PHx_48h group no matter in overall pseudo-bulk levels or in various cell types, especially hepatocytes. The mRNA expression levels of 17 genes were also up-regulated in PHx_48h group (Supplementary Figure 5). And the regeneration 17-gene signature was up-regulated in cluster7 of hepatocytes (Figure 4D, 4E). Further, we performed the pseudotime analysis of hepatocytes using monocle2 pipeline. Of note, hepatocytes were ordered into three branches/states and followed with the pseudotime from PHx_0h to PHx_48h (Figure 4F). Concordantly, the distribution of cluster7 and regeneration-related hepatocytes were also mainly in the end position of the branches (Figure 4G). Then, we estimated the expression of 17 genes following the pseudotime. Result also indicated that the higher expression levels of 17 genes, the latter the pseudotime (Figure 4H).

### Transcriptional factor network and pseudotime analysis revealed the potential regulatory regulons and the communication differences during regeneration

**Figure 5:**
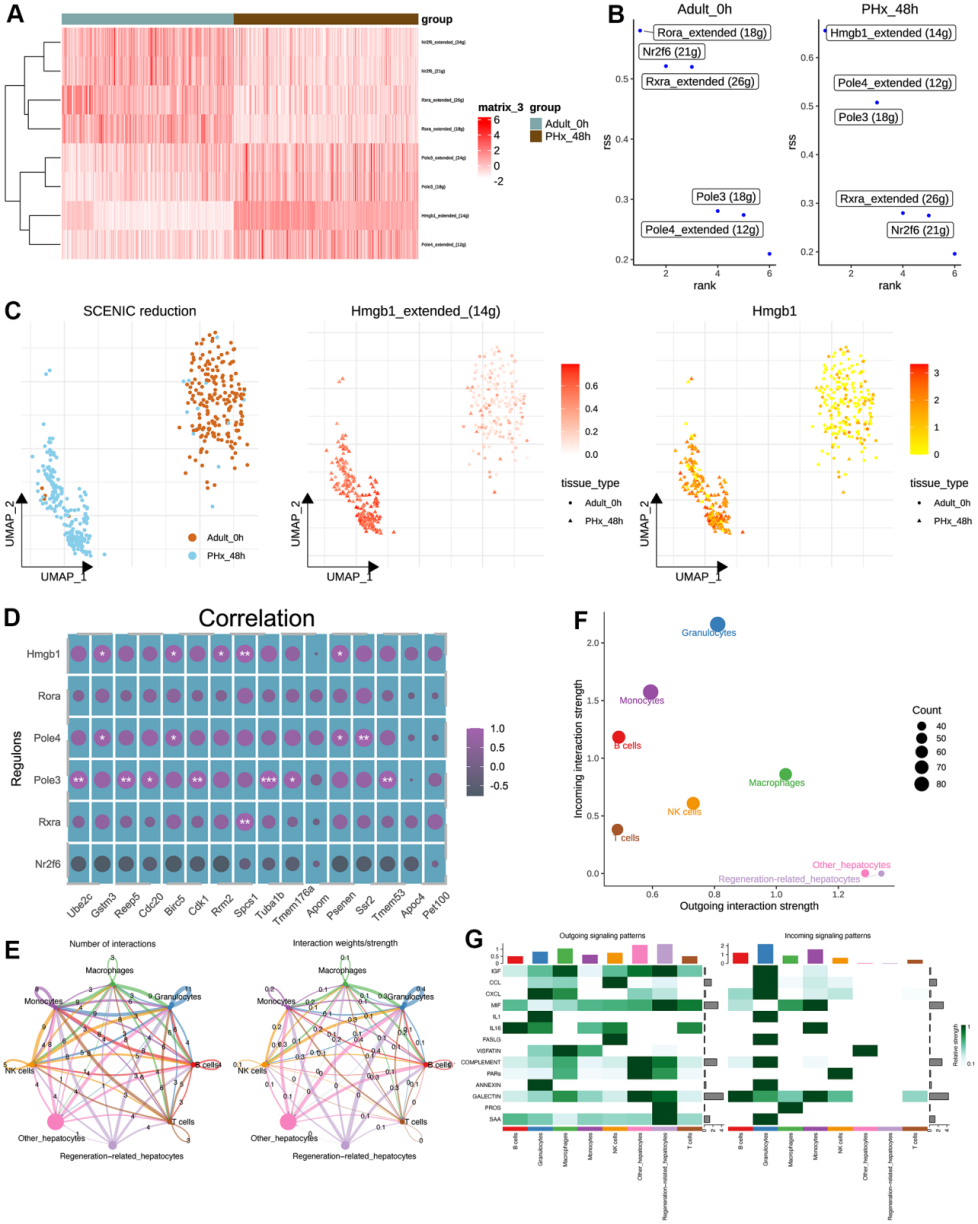
**Transcriptional regulatory network and cell-cell communication analyses of hepatocytes.** (**A**) Heatmap shows the differences of TFs in hepatocytes between the PHx 0h and PHx 48h groups. Only 400 hepatocytes were randomly chosen for SCENIC analysis. (**B**) Top activities of TFs between different groups of hepatocytes. RSS indicates Regulon Specificity Score. (**C**) Activity distribution of Hmgb1 and expression distribution of Hmgb1 in hepatocytes. (**D**) Correlation analysis of SCENIC-identified regulons and 17 machine-learning-identified feature genes in bulk RNA-seq of PHx samples. (**E**) Cellchat analysis of all cell types. Both interaction numbers and interaction strengths were showed. (**F**) Scatter plot indicates the differences of incoming and outgoing interaction strengths among all cell types. (**G**) Top cell cytokines were showed in heatmap across all cell types in PHx.

The good performance of the constructed regeneration model indicates the gene expression varied largely during PHx. Therefore, we adopted SCENIC pipeline [21] for further uncovering the differences of transcriptional factors. As a result, several TFs were found out (Figure 5A). According to the calculated Regulon Specificity Score (RSS), The top TFs were different from the two groups, including Hmgb1, Rora, Pole4, Pole3, Rxra and Nr2f6 (Figure 5B). According to the inferred TFs, we further performed the reduction analysis and result implied the different distribution of regeneration-related hepatocytes and other hepatocytes from PHx_0h and PHx_48h groups (Figure 5C). And the mRNA expression level and inferred activity score of Hmgb1 were both higher in regeneration-related hepatocytes rather than other hepatocytes (Figure 5C). Therefore, we inferred our that Hmgb1 might be vital in the regeneration-related hepatocytes of PHx_48h group. Then, we performed the correlation analysis of the inferred TFs and 17 regeneration-related genes. Results showed that Hmgb1 and Pole3 had significant correlated with various gens (Figure 5D).

Parallelly, the cell-cell communication analysis was also performed using cellchat pipeline [20]. Collectively, the overall communication ability with other non-parenchymal cell types was still strong during the regeneration process (Figure 5E). Of interest, we found that the outgoing interaction strength, indicating the secretory ability, of regeneration-related hepatocytes was stronger than other hepatocytes (Figure 5F), including IGF, MIF and SAA signaling (Figure 5G).

### Validation of the regeneration-related signature in seven external cohorts

**Figure 6:**
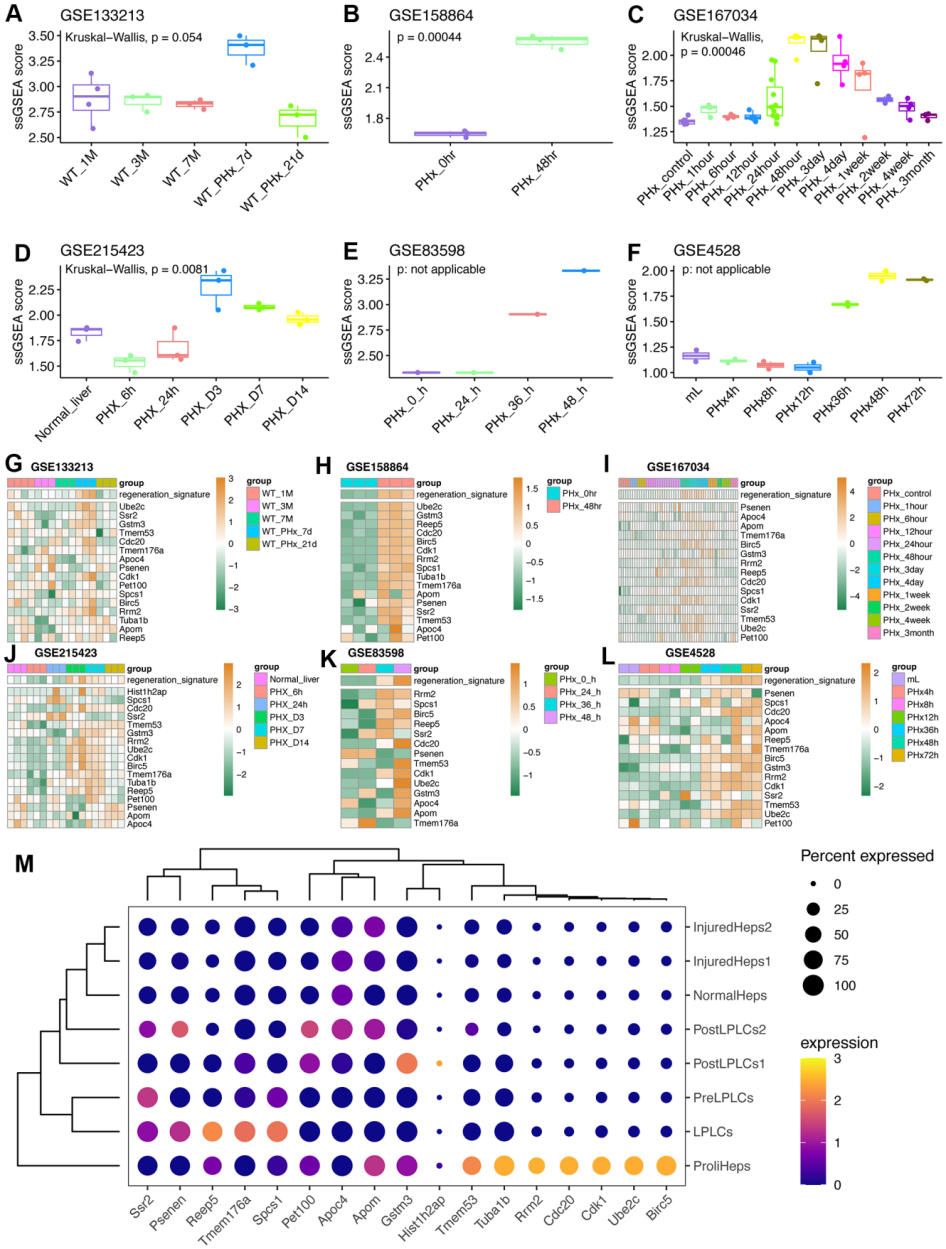
**Validation of the regeneration-related signature in external datasets related to PHx.** (**A**) Regeneration-related signature score in the GSE133213 dataset. WT: wild-type mouse. (**B**) Regeneration-related signature score in the GSE158864 dataset. The data only contained samples at 0-hour and 48-hour after PHx. (**C**) Regeneration-related signature score in the GSE167034 dataset related to multiple timepoints after PHx surgery. (**D**) Regeneration-related signature score in the GSE215423 dataset containing samples at 6h, 24h, Day3, Day7 and Day14 after PHx in mice. (**E**) Regeneration-related signature score in the GSE83598 dataset. Each timepoint after PHx only had one sample. (**F**) Regeneration-related signature score in the GSE4528 dataset. (**G**) Expression levels of 17 feature genes in GSE133213 dataset. (**H**) Expression levels of 17 feature genes in GSE158864 dataset. (**I**) Expression levels of 17 feature genes in GSE167034 dataset. (**J**) Expression levels of 17 feature genes in GSE215423 dataset. (**K**) Expression levels of 17 feature genes in GSE83598 dataset. (**L**) Expression levels of 17 feature genes in GSE4528 dataset. Undetected genes were not showed. (**M**) Dot plot revealing the expression levels of 17 genes in DDC model from GSE212692 dataset. LPLC: liver progenitor-like cells; DDC: 3,5-diethoxycarbonyl-1,4-dihydrocollidin.

After the comprehensive exploration of the regeneration-related hepatocytes, we tried to better understand the regeneration-related signature we identified. For that sake, we downloaded six datasets from GEO database, which were related to PHx at different timepoints, including GSE133213, GSE215423, GSE83598, GSE4528, GSE167034 and GSE158864 (Table 1). Intriguingly, we found that the regeneration score will be gradually up-regulated and gradually down-regulated during the PHx and liver recovery (Figure 6A–6F). And at the time point of 48h the signature score will reach the peak (Figure 6B, 6C, 6E, 6F). More than this, we also draw out the expression of the 17 genes and the corresponding ssGSEA-calculated signature score in all these six datasets (Figure 6G–6L). Interestingly, we found that most gene fitted the expression pattern of the signature score, especially Birc5. Finally, we also detected the expression levels of 17 genes in scRNA-seq data from liver progenitor-like cells (LPLC) to mature hepatocytes (Figure 6M). Although the murine model was liver injure induced by 3,5-diethoxycarbonyl-1,4-dihydrocollidin (DDC) treatment, most of 17 genes were up-regulated in progenitor-like cells (LPLC) and proliferative hepatocytes, partly revealing the activity of the regeneration signature score.

### Functional analysis indicated the involvement of Birc5 in cell cycle and liver regeneration

**Figure 7:**
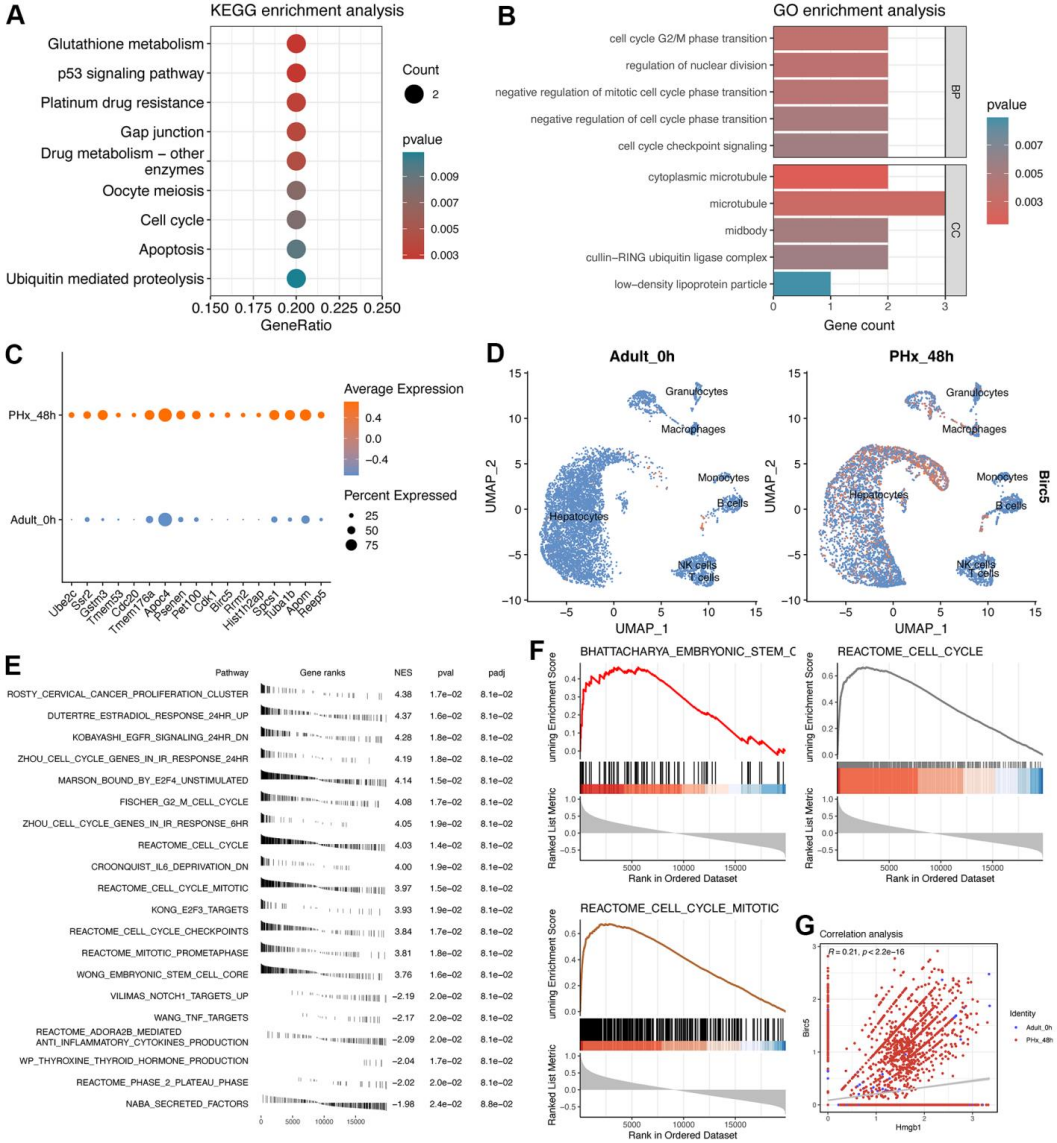
**Enrichment analyses of 17-signature-related genes and GSEA analysis of Birc5.** (**A**) KEGG enrichment analysis of 17 regeneration-signature-related genes. Only significant terms were showed and the red color represents lower P value. (**B**) GO enrichment analysis of 17 regeneration-signature-related genes, showing in bar plot. BP indicates biological process while CC indicates cellular component. (**C**) Average expression of the 17 genes between PHx 0h and PHx 48 groups, which was displayed using dot plot. (**D**) Distribution of Birc5 expression in all cell types between PHx 0h and PHx 48 groups. (**E**) fGSEA analysis of Birc5 and only top significant terms were listed. (**F**) GSEA analysis of Birc5 gene. The method for ranking detected genes was according to the correlation coefficients with Birc5 gene, which could make us to be capable of enriching Birc5-related terms. (**G**) correlation analysis between birc5 and Hmgb1.

Subsequently, we conducted the GO and KEGG analyses of the 17 genes (Figure 7A, 7B). Notably, the proliferation-related pathways, including “p53 signaling pathway”, “Cell cycle” and “Regulation of nuclear division” were highly enriched. Then, we explored the average expression level of the 17 genes from single cell data (Figure 7C), especially Birc5 (Figure 7D). Then, GSE167034 dataset was used to run the GSEA analysis of Birc5, which could be used to better understand the potential function of Birc5. Interestingly, result showed that Birc5 had been highly correlated with the “Reactome_cell_cycle” pathway and stem cell-related pathways, consistent with the enrichment analysis of 17 genes (Figure 7E, 7F). Finally, we performed the correlation analysis between Birc5 and Transcriptional factor Hmgb1, which also highlighted the expression correlation between them (Figure 7G).

### Loss of spatial characteristics in hepatocytes during regeneration process

**Figure 8:**
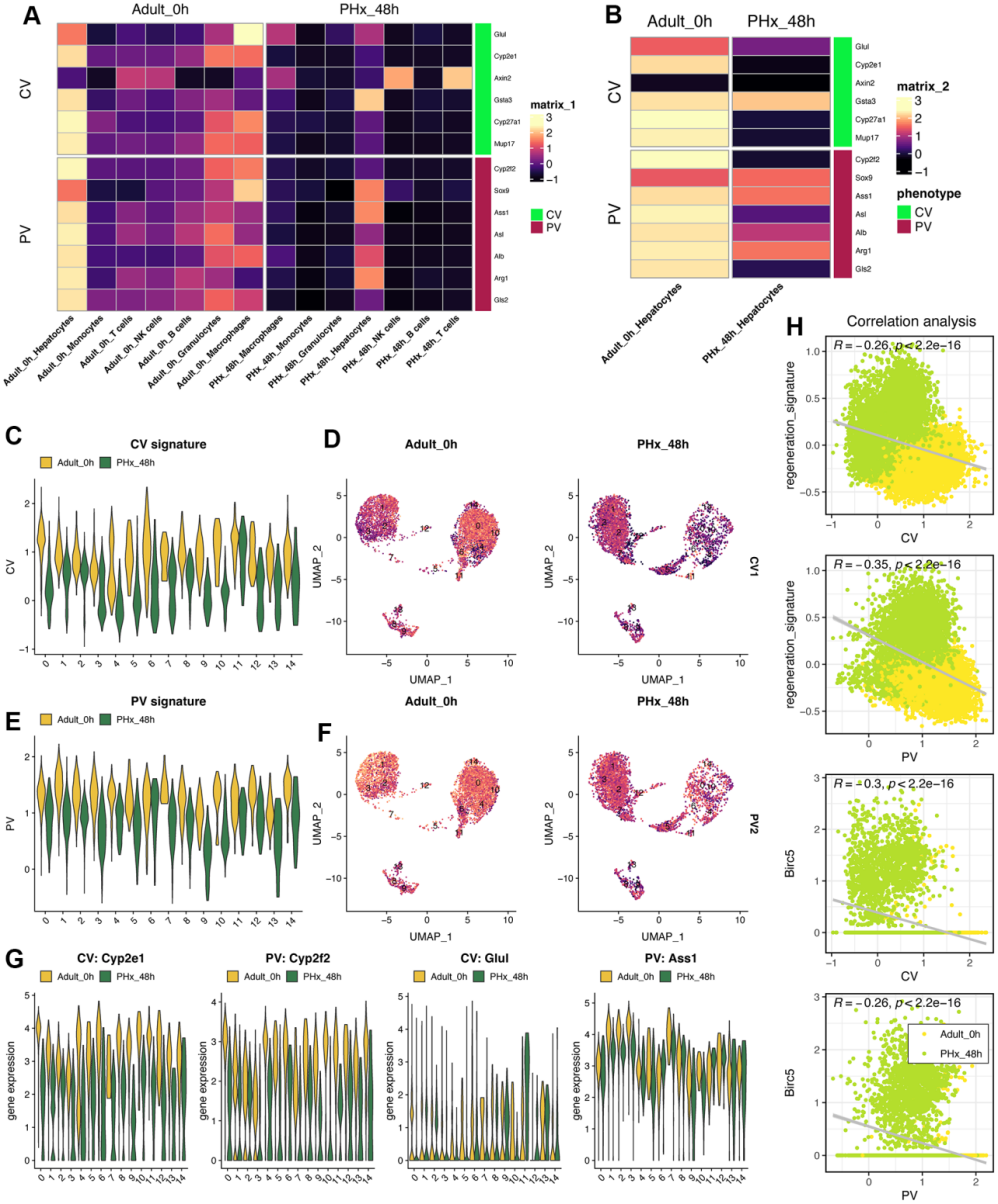
**Characteristics of liver zonation in mice after PHx.** (**A**) Expression levels of liver zonation genes across all cell types at PHx 0h and PHx 48h groups. PV: periportal vein area; CV: pericentral vein area. (**B**) Expression levels of liver zonation genes in hepatocytes with the comparison between the PHx 0h group and the PHx 48h group. (**C**) Violin plot shows the CV signature score in all 15 hepatocytes clusters. (**D**) Distribution of the CV signature score in all 15 hepatocytes clusters. The CV signature score was calculated using “addmodule” function in seurat package. (**E**) Violin plot shows the PV signature score in all 15 hepatocytes clusters. (**F**) Distribution of the PV signature score in all 15 hepatocytes clusters. The PV signature score was calculated using “addmodule” function in seurat package. (**G**) Expression levels of Cyp2e1, Cyp2f2, Glul and Ass1, which were typical biomarkers of PV or CV, in all hepatocyte clusters. Yellow indicates the PHx 0h group while Green indicates the PHx 48h group. (**H**) Correlation analyses among regeneration signature, CV signature, PV signature and Birc5 gene in all single hepatocyte cells from both PHx 0h and 48h groups.

Spatial characteristics of hepatocytes indicates the maturation and biological function of hepatocytes. Herein, we collected the genes/biomarkers related to Portal Vein area (PV) and Central Vein area (CV), including Cyp2e1, Cyp2f2, Glul, Ass1 and so on (Figure 8A). Result indicated that the expression of PV- or CV-related genes will be down-regulated in almost all cell types (Figure 8A), especially hepatocytes (Figure 8B). Then, we utilized the “addmodule” function in Seurat packages to further calculate the overall signatures of PV and CV in all 15 hepatocyte clusters (Figure 8C–8F). Obviously, the PV and CV signature score will decrease when the hepatocytes tried to change its status from quiescent state into proliferative state (Figure 8E, 8F). The landmark genes, including Cyp2e1, Cyp2f2, Glul and Ass1, also supported the results that almost all clusters of hepatocytes lose the spatial characteristics to some extent, no matter PV or CV (Figure 8G). Notably, we also found that the identified regeneration signature and Birc5 were both negatively correlated with both PV and CV signature, respectively (Figure 8H).

### Validation of the regeneration-related signature post PHx, especially Birc5

**Figure 9:**
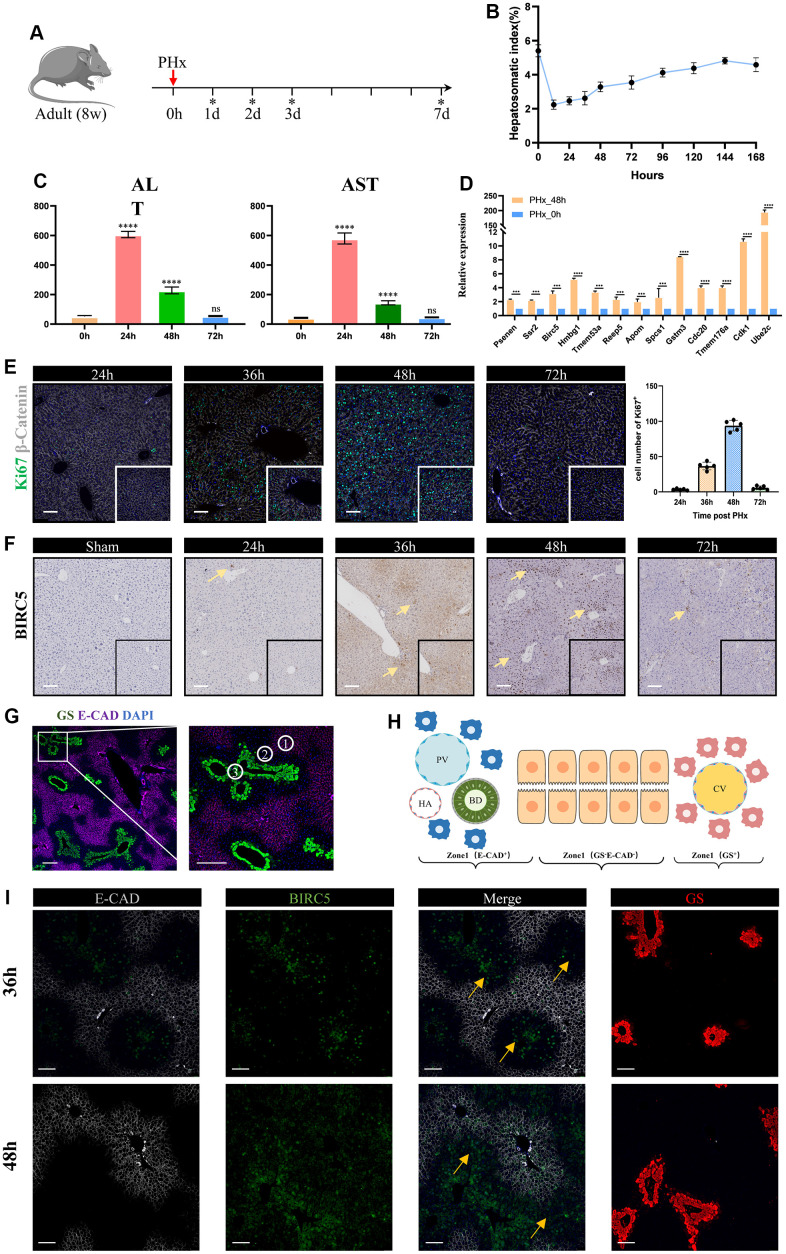
**Validation of liver regeneration-related signature post PHx.** (**A**) A schematic of experimental design procedure: Mice underwent surgical resection. Ticks show the time point when mice were sacrificed. Liver tissues are harvested at 24h, 36h, 48h, 72h and 7days. (**B**) Time curve showed the liver-to-body weight of remanent liver after PHx. Data are mean±sd, ****p<0.0001, ns: no significant, (n=3 mice per groups). (**C**) The levels of serum indicate that liver mass return to normal function after 3days post PHx. (**D**) Relative expression levels of liver regeneration-related genes in hepatocytes between the PHx 0h group and the PHx 48h group. Data are “mean±sd”, ***p<0.001, ****p<0.0001. (**E**) Representative immunofluorescence shows multiplication capacity of hepatocytes at different time point post PHx, Ki67 (green), β-catenin (gray), DAPI (blue), Scale bar=100μm. Graphical representation indicates that the 36–48-hour time period represents an active mitotic phase. (**F**) We performed immunohistochemical imaging to evaluate the expression of Birc5 in both post-PHx and Sham livers. The significantly expressed hepatocytes were indicated by yellow arrows, scale bar=100μm. (**G**) Co-staining of zonal specific markers during homeostasis. Glul, which encodes glutamine synthetase (GS), was exclusively expressed in pericentral hepatocytes, while Cdh1, which encodes E-cadherin, was expressed only in periportal hepatocytes. (**H**) schematic of liver zonation. (**I**) Spatial distribution of Bircs5 following PHx at 36h and 48h revealed that liver regeneration-related hepatocytes primarily initiated in zone 2 before proceeding to other regions.

**Figure 10:**
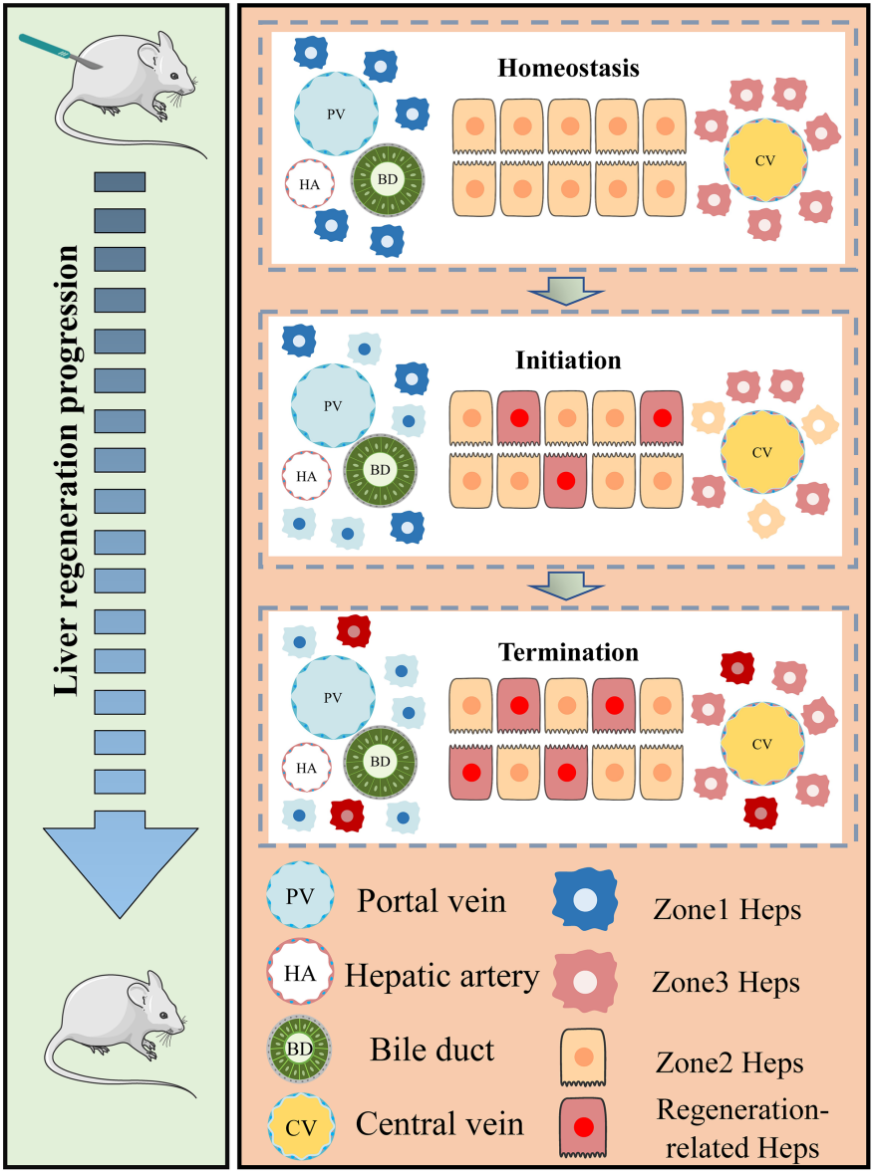
**Regional characteristics of live regeneration-related hepatocytes during acute liver injury.** The left panel of our study demonstrates the process of liver regeneration following PHx. The surgical resection of liver tissue triggers the rapid re-entry of remnant hepatocytes into the regeneration state. The right panel summarizes the zonal variation in regeneration-related hepatocytes during acute liver injury (AIL). Under homeostatic conditions, hepatocytes in different zones remain in a quiescent state. Portal vein (PV) and central vein (CV) hepatocytes are located at both ends of the hepatic lobules and exhibit distinct spatial gene expression patterns. However, during liver damage, newborn hepatocytes initiate in the midlobular regions before progressing towards the periportal and pericentral areas. At the same time, zonal differences in PV and CV hepatocyte gene expression decrease as hepatocytes respond to the regenerative challenge (hepatocytes with blue and yellow dim out). During the termination phase, newborn hepatocytes repopulate different regions, leading to the recovery of liver function.

We constructed a PHx mouse model, and mouse liver tissues were harvested at 24h, 36h, 48h, 72h and 7days after PHx (Figure 9A, 9B). The levels of serum indicate that liver mass return to normal function after 3days post PHx (Figure 9C). qRT-PCR assays showed that all 17 regeneration-related genes were up-regulated in 48h-hepatocytes compared to the PHx 0h group (Figure 9D, 9E). In addition, we performed immunohistochemical imaging to evaluate the expression of Birc5 in both post-PHx and Sham livers and consistent result was obtained (Figure 9F). Spatial distribution of Birc5 following PHx at 36h and 48h revealed that liver regeneration-related hepatocytes primarily initiated in zone 2 before proceeding to other regions (Figures 9G–9I, 10).

## DISCUSSION

Liver transplantation is the only effective treatment for patients with end-stage liver diseases, however, the current supply of liver organ was not enough to cover patient need [23]. Therefore, understanding the mechanism of hepatic proliferation and finding ways to promote hepatocyte growth *in vivo* after transplantation are urgently needed. Cells related to the hepatic lineage have great potential in the treatment of end-stage liver diseases. Recent studies have demonstrated that cell plasticity and epigenic remodeling that occur in hepatocytes during liver damage repair. Through chromatin remodeling, a proportion of quiescent hepatocytes ultimately transformed into proliferative state, generating transcriptomic alterations which allows cells to re-enter the cell cycle [24]. Utilizing scRNA-seq techniques, we generated an unbiased and comprehensive cell atlas related to liver regeneration. We collected liver tissue samples from normal mice and mice 48 hours following PHx surgery to annotate different cell subsets and reveal changes in the transcriptome of hepatocytes, as well as illustrate their interactions during liver regeneration. This study provides a critical foundation for further understanding the reprogramming effects and molecular mechanisms of mature hepatocytes during liver regeneration. regeneration. Numerous studies and several pioneer factors, such as EGFR, IL-6, TGFβ revealed that liver regeneration and tumor development are inseparable process [11, 25, 26]. Of note, a signature of a hepatocyte subset related to liver regeneration was demonstrated to be useful in determining the regenerative capacity of hepatocytes, particularly Birc5, indicating that it may be a promising key regulatory factor. Moreover, The Birc5 have also been confirmed during rat PHx liver injury models and increased during 5-7days after human transplantation [27], partly indicating the reliability of our identified signature and the crucial of Birc5 for hepatocyte proliferation and regeneration in PHx.

Under normal circumstances, the liver is a relatively stationary organ, and mature hepatocytes are largely quiescent. Random DNA-labeling revealed that only 1.9% hepatocytes divided more than once in 8 to 10 months [28]. However, the liver possesses an exceptional ability to regenerate rapidly following partial hepatectomy, enabling surgical resection of a portion of the liver as well as living donor and segmented liver transplantation [29]. Recent evidence suggests that hepatocytes proliferate randomly in all hepatic regions to maintain homeostasis [30]. In the process from partial hepatectomy (PHx) to regeneration, the hepatocyte lineage tracing studies showed that approximately 98% of newly produced hepatocytes were derived from pre-existing hepatocytes, with the remaining fraction likely to be derived from pre-existing hepatic progenitor cells [31]. Particularly, the obtained non-parenchymal liver cells will increase at 48h after PHx both in the two 48h scRNA-seq samples, indicating the involvement of cell-cell communication during regeneration. Advancement in the field of liver regeneration is potential role of macrophage during inflammation and reprogramming process [11, 32]. Recently, Li et al. has reported that hepatocytes could be triggered and induced into proliferative and regenerative state by Kupffer-cell-derived IL-6 after suffering injury [11]. Thus, understanding the mechanism of hepatocyte regeneration, mastering the process of hepatocyte proliferation from static to dynamic, and identifying the regeneration state of hepatocytes are conducive to better culturing hepatocytes *in vitro* and determining the state of hepatocytes after transplantation into damaged livers.

In this work, we screened and obtained a 17-gene model using hdWGCNA, univariate analysis, multivariate analysis, LASSO analysis, and seven other machine learning analyses, including genes of Ube2c, Ssr2, Gstm3, Tmem53, Cdc20, Tmem176a, Apoc4, Psenen, Pet100, Cdk1, Birc5, Rrm2, Hist1h2ap, Spcs1, Tuba1b, Apom and Reep5. The process of hepatocyte proliferation from static state to dynamic state often needs to enter proliferation cycle. Herein, it can be seen that some cycle-related genes, such as Cdk1 and Cdc20, are included in this model. Using enrichment analysis, we also found that these 17 genes were closely related to the cycle. In particular, GSEA and fGSEA analyses of the Birc5 gene revealed that Birc5 was strongly associated with cell cycle. More interestingly, in several external validation datasets, the score levels of Birc5 and 17 genes were highly consistent, better reflecting the proliferative status of regeneration-related hepatocytes, and seemed to be a relatively reliable biomarker/indicator. Another fascinating part in the field of liver regeneration is liver zonation, which remain several unsolved problems and several unanswered questions remain regarding how liver zonation can drive hepatic pathophysiology. Our single-cell analysis revealed that zonal differences of hepatocytes gene expression decrease, which is consistent with previous results about liver regeneration post PHx [7]. Previous studies with different lineage tracing strategies came up with widely varying conclusion [33, 34]. Our work revealed that midlobular hepatocytes were primary involved in acute liver injury and further studies are needed to explore how liver zonation affect liver regeneration-related hepatocytes and its role in spatial compartmentalization.

Survivin, also known as Birc5 and as part of the chromosomal complex during mitosis, directs the aggregation and segregation of chromosomes as well as cytoplasmic division [35]. When it occurs at the interphase, it is mainly located in the cytoplasm and participates in the anti-apoptotic function of cells [35]. Another finding suggests that survivin may be regulated by metabolic stress, which is consistent with the significant metabolic changes observed following PHx [36]. This work emphasized that Birc5 can act as a potential marker for estimating hepatocyte entering the peak of proliferation, with a significant negative correlation with metabolic zonation of liver, suggesting that it plays an important role in the process of liver regeneration and disruption of liver zonation.

As transcription factors are an important regulatory factor for cell fate determination [37], we tended to explore regulatory factors for triggering the static hepatocytes into proliferative and regenerative hepatocytes. Therefore, SCENIC pipeline was adopted and we found that Hmgb1 might be a potential target for hepatocyte-based regenerative medicine. The high mobility group box 1 gene (Hmgb1) encodes a very abundant non-histone nuclear protein. Hmgb1 is not only a nuclear factor that enhances transcription but also an important cytokine that mediates the pathological effects of cancers, sepsis and other diseases [38]. Hmgb1 was typical and known to be participated in immune response and inflammation [38]. Recently, a study shows that IL6, another known proinflammatory factor, can be secreted by Kupffer cells and get involved in the reprogramming and regeneration of hepatocytes in liver injury via STAT3 pathway [11]. Using the sequencing data from this study, we also found that the identified genes were mainly expressed in proliferative hepatocytes and liver progenitor-like cells (LPLC), partly validating the reliability of the signature.

In this study, we utilized scRNA-seq data to identify key cell subsets and their associated specific signatures during liver regeneration following PHx. Furthermore, we sought to determine whether these signatures could be applicable to other forms of liver injury. After analyzing published scRNA-seq data regarding the expression of Birc5 and Hmgb1, we found that hepatocyte reprogramming was likely closely linked to these genes. It is noteworthy to consider whether Birc5 and Hmgb1 may also play important roles in other types of liver injury.

## CONCLUSIONS

In conclusion, our study has identified a regeneration-related signature that can be used to estimate liver regeneration. These findings will contribute to a better understanding and interpretation of the biological and clinical aspects of liver-regenerating hepatocytes, providing a fundamental basis for the development of effective diagnosis and therapy. The genes identified in this study, especially Birc5, may be used to evaluate the liver state of patients with liver failure. Alternatively, clinicians could use these molecules as biomarkers to predict the likelihood of spontaneous liver regeneration in patients.

## Supplementary Material

Supplementary Figures
