# Supplementary material for: Machine learning and single cell RNA sequencing analysis identifies regeneration-related hepatocytes and highlights a Birc5-related model for identifying cell proliferative ability
Source: Aging (Albany NY). 2023 Jun 14;15(11):5007–31. doi: 10.18632/aging.204775 (PMC10292894; doi:10.18632/aging.204775)
Supplement: Supplementary Figures [file aging-15-204775-s001.pdf]

## SUPPLEMENTARY FIGURES

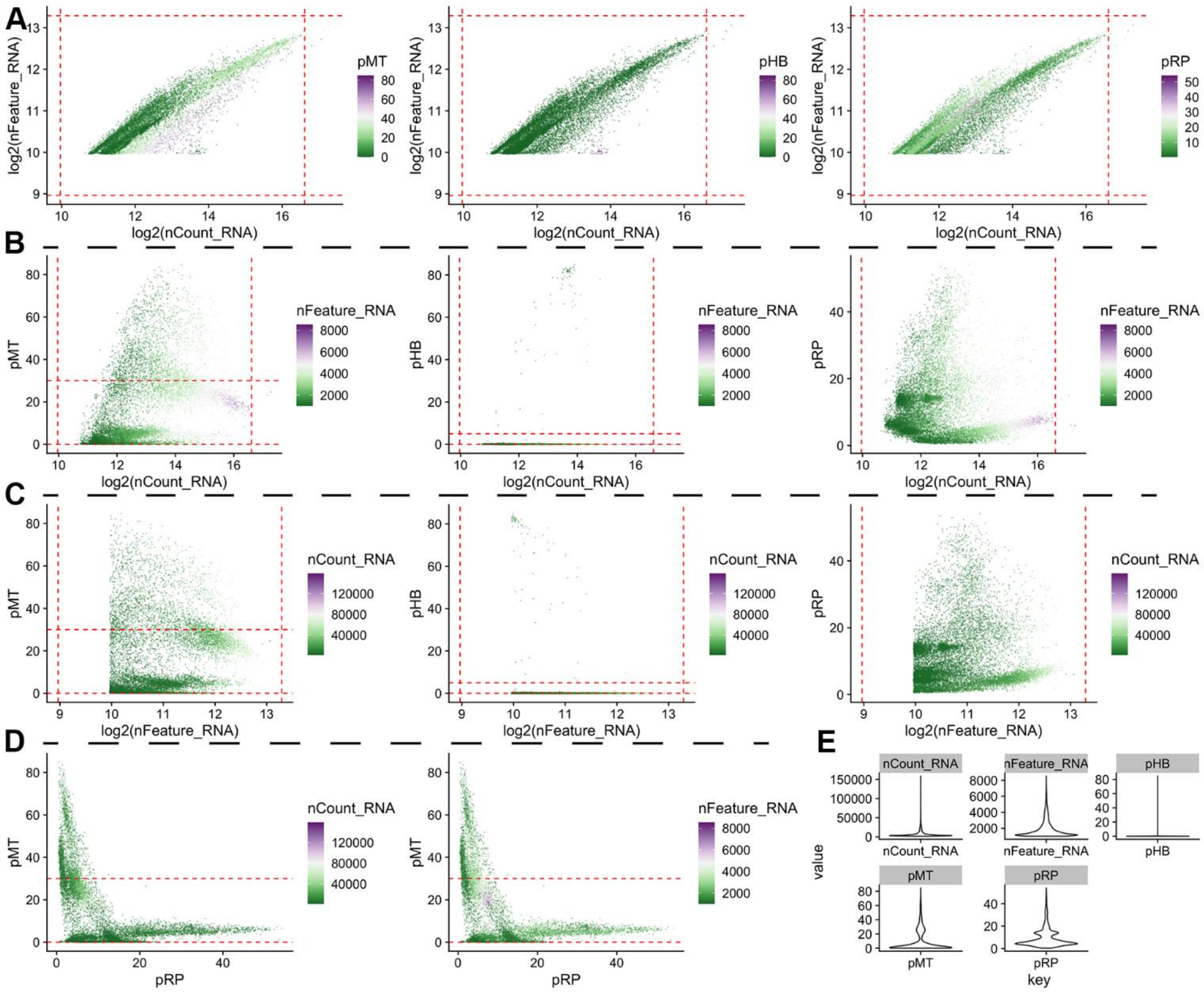


**Supplementary Figure 1. Parameters related to detected cells before quality control.** (**A**) Scatter plots show the levels of total features, total counts of single cells. Color represents the percentages of mitochondria genes (pMT), hemoglobin genes (pHB), ribosomal protein genes (pRP), respectively. (**B**) Total detected genes levels (nFeature_RNA) in scatter plot. Redline represents the reasonable ranges of quality cells. (**C**) Total detected counts levels (nCount_RNA) in scatter plot. Redline represents the reasonable ranges of quality cells. (**D**) Levels of nFeature_RNA and nCount_RNA in Scatter plot. Ordinate axis is pMT and horizontal axis is pRP. (**E**) Violin plot showing the levels of five parameters, including nCount_RNA, nFeature_RNA, pHB, pMT and pRP.

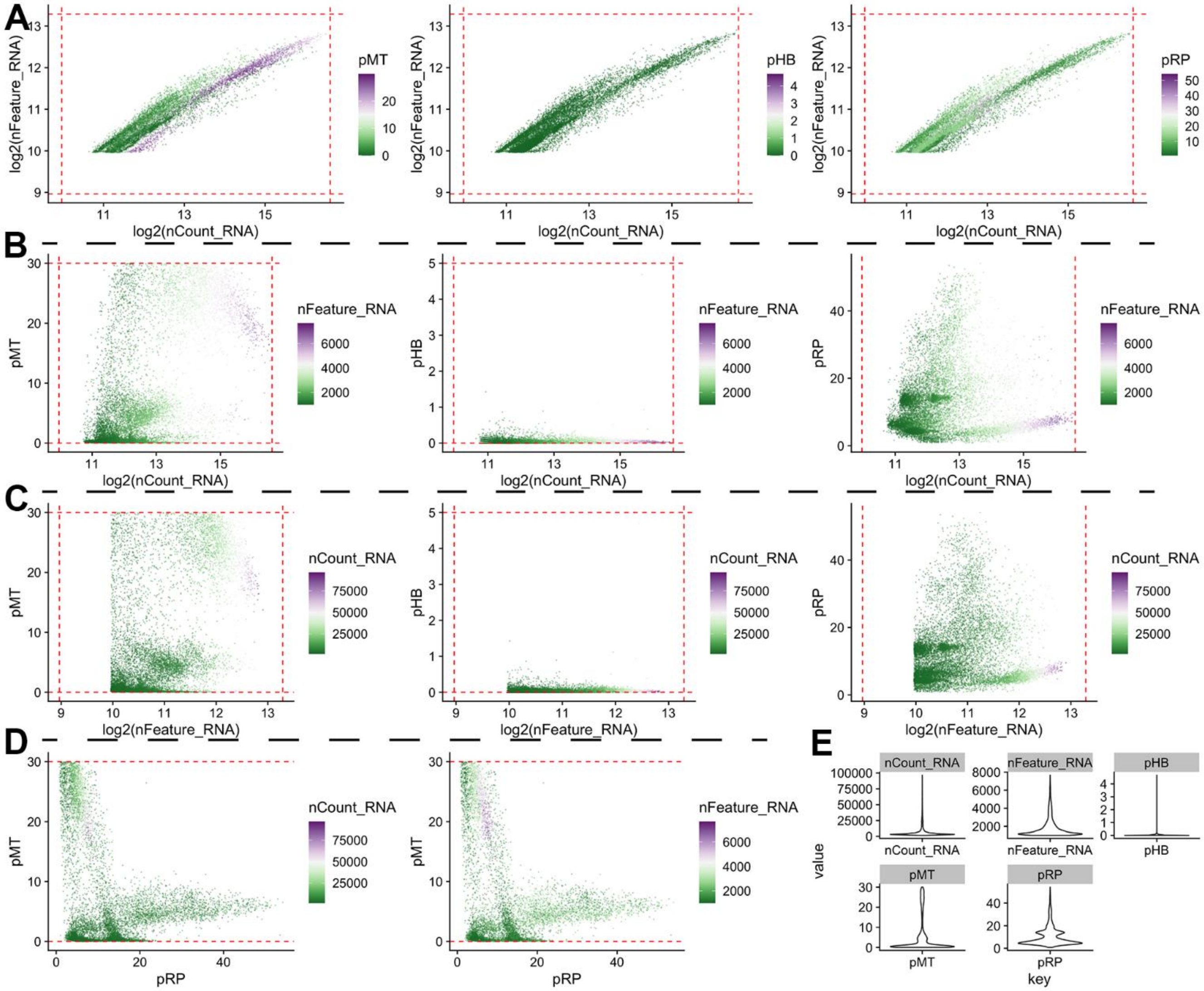


**Supplementary Figure 2. Parameters related to detected cells after quality control.** (**A**) Scatter plots show the levels of total features, total counts of single cells after stringent quality control. Color represents the percentages of mitochondria genes (pMT), hemoglobin genes (pHB), ribosomal protein genes (pRP), respectively. (**B**) Total detected genes levels (nFeature_RNA) in scatter plot after stringent quality control. Redline represents the reasonable ranges of quality cells. (**C**) Total detected counts levels (nCount_RNA) in scatter plot after stringent quality control. Redline represents the reasonable ranges of quality cells. (**D**) Levels of nFeature_RNA and nCount_RNA in Scatter plot after stringent quality control. Ordinate axis is pMT and horizontal axis is pRP. (**E**) Violin plot showing the levels of five parameters, including nCount_RNA, nFeature_RNA, pHB, pMT and pRP.

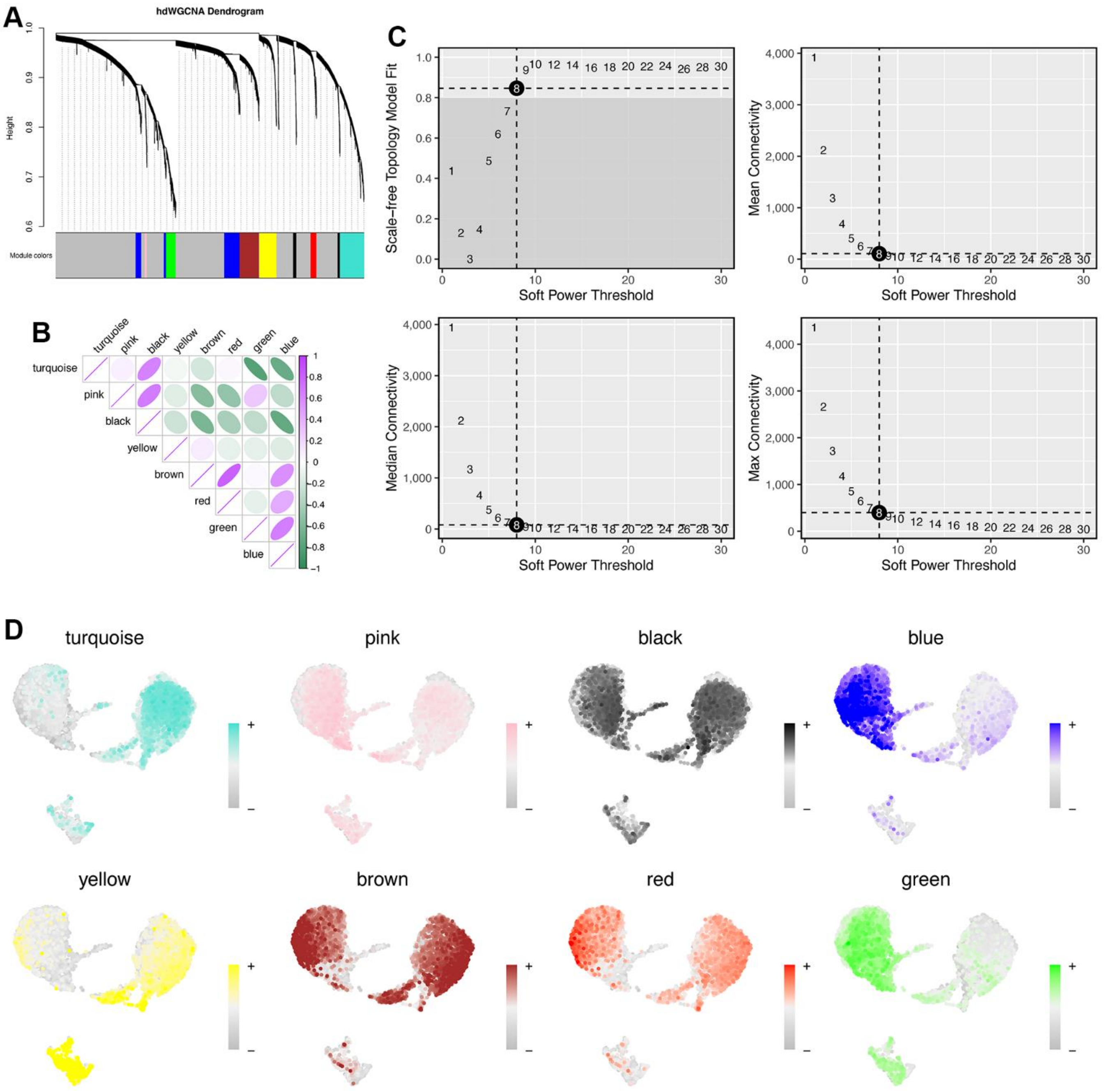


**Supplementary Figure 3. High dimensional WGCNA pipeline for hepatocytes.** (**A**) The hdWGCNA dendrogram of hepatocytes. (**B**) Correlation analysis between all identified modules by hdWGCNA. (**C**) Selection of soft power for running hdWGCNA. Max, median and mean connectivity were showed, respectively. (**D**) Distribution of module scores in hepatocytes. There are total eight gene modules for hepatocytes.

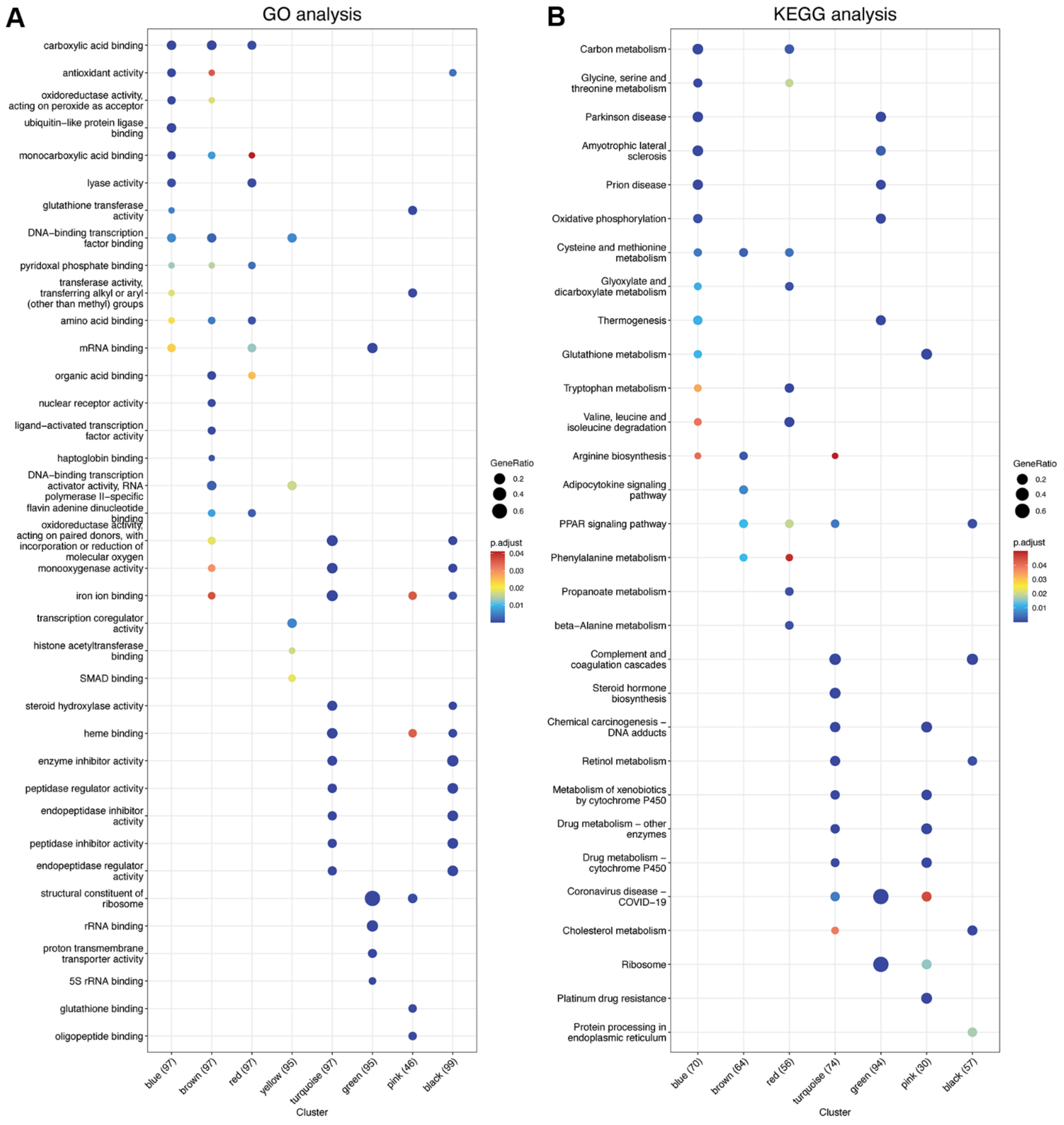


**Supplementary Figure 4.** Functional analyses for all modules using GO (**A**) and KEGG databases (**B**). Only significant terms were showed.

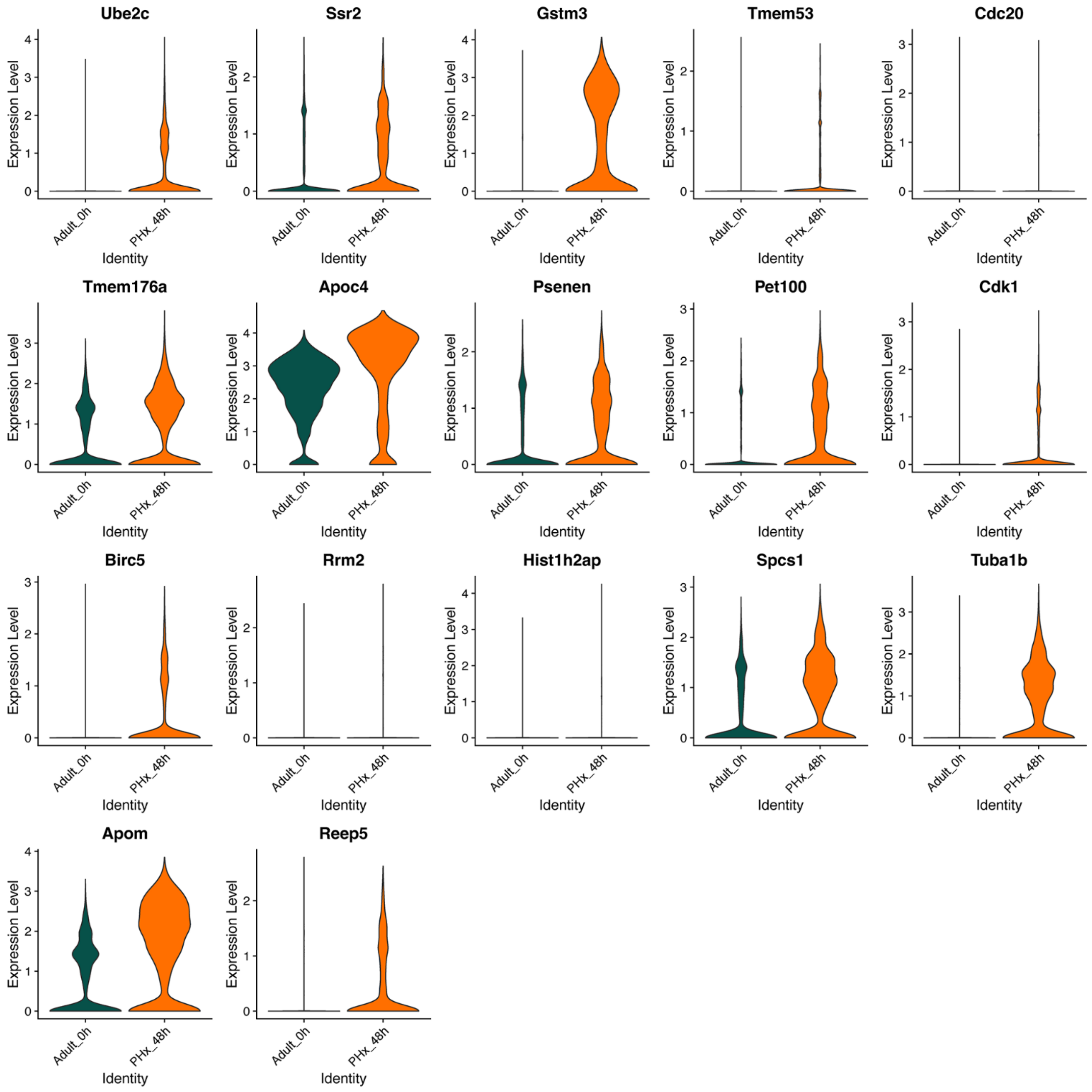


**Supplementary Figure 5. Expression of 17 regeneration-related genes in scRNA-seq data related to PHx.**
